# Effects of Intestinal Microbiota on Brain Development in Humanized Gnotobiotic Mice

**DOI:** 10.1038/s41598-018-23692-w

**Published:** 2018-04-03

**Authors:** Jing Lu, Lei Lu, Yueyue Yu, Joanne Cluette-Brown, Camilia R. Martin, Erika C. Claud

**Affiliations:** 1 The University of Chicago, Pritzker School of Medicine, Department of Pediatrics, Chicago, IL 60637 USA; 2 Beth Israel Deaconess Medical Center, Division of Gastroenterology, Boston, MA 02215 USA; 3 Beth Israel Deaconess Medical Center, Harvard Medical School, Department of Neonatology and Division of Translational Research, Boston, MA 02215 USA

## Abstract

Poor growth in the Neonatal Intensive Care Unit is associated with an increased risk for poor neurodevelopmental outcomes for preterm infants, however the mechanism is unclear. The microbiome has increasingly been recognized as a modifiable environmental factor to influence host development. Here we explore the hypothesis that the microbiome influences both growth phenotype and brain development. A germ free mouse transfaunation model was used to examine the effects of preterm infant microbiotas known to induce either high growth or low growth phenotypes on postnatal brain development. The microbiome which induced the low growth phenotype was associated with decreases in the neuronal markers NeuN and neurofilament-L as well as the myelination marker MBP when compared to the microbiome associated with the high growth phenotype. Additionally, poor growth phenotype-associated microbiota was associated with increased neuroinflammation marked by increased *Nos1*, as well as alteration in IGF-1 pathway including decreased circulating and brain IGF-1, decreased circulating IGFBP3, and increased *Igfbp3* brain mRNA expression. This study suggests that growth-associated microbiota can influence early neuron and oligodendrocyte development and that this effect may be mediated by effects on neuroinflammation and circulating IGF-1.

## Introduction

Around the time of birth and thereafter, newborns are rapidly colonized with a community of microbes that starts the process of gut microbiota assembly and continues during the first 2–3 years of life^1^. The early assembly of microbiota has been shown to shape host development of the gut^2,3^, endocrine system^4^, lung^5^, liver^6,7^, bone^8^ and brain^1,9,10^. More specifically, this window of early gut microbiota development parallels nervous system development across prenatal and early postnatal stages. Brain development such as neurogenesis, neuronal migration, axonal and dendritic growth, synaptogenesis, and myelination begins early in prenatal life and continues postnatally^10,11^. The concurrent, early, postnatal window of both microbiome and brain development is a critical time period for investigation to determine whether an interplay between these developmental processes exists.

Preterm infants, particularly those born at less than 32-week of gestational and/or with a birth weight less than 1500 g, are at risk for adverse neurological outcomes including later cognitive and behavioral deficits^12,13^. These deficits are considered to be the consequence of complications associated with prematurity which impairs brain development^14^. Furthermore, while critical development for full term infants occurs *in utero* under limited influence of microbiota, for preterm infants it largely occurs *ex utero* shaped by environmental influences including the development of the microbiome. The microbiome of the preterm infant is shaped by the NICU environment including instrumentation, frequent antibiotic use, multiple care givers, and a hospital environment. The combination of postnatal insults and developmental immaturity of both the gut microbiota and the nervous system makes preterm infants a unique population in which to study the impact of initial microbiota colonization and development on brain development.

Recently, our group demonstrated that germ free (GF) mice colonized with two different human preterm infant fecal samples yielded distinct growth phenotypes^15^. We and others have previously demonstrated that the microbiome of preterm infants changes over time with distinct clustering at less than 2 weeks of life^16,17^. Thus, to study the effects of these early microbial colonization patterns on host development, samples chosen for transfaunation were from human preterm infants prior to two weeks of life with different growth rates. Our data showed that a preterm infant growth phenotype could be transferred to mice via the microbiota. Mice colonized with microbiota from a preterm infant with good growth (>10 gm/k/day weight gain, Microbiota _PRETERM INFANT_-High (M_PI_-H)) had significantly more weight gain (20%) postnatally than mice colonized with microbes from a preterm infant with poor postnatal growth (<10 gm/k/day weight gain, Microbiota _PRETERM INFANT_-Low (M_PI_-L)). We demonstrated that M_PI_-L mice had increased intestinal inflammation using nuclear factor-kappa beta (NF-κB) activation as a marker and by investigating production of NF-κB-mediated inflammatory cytokines at both the intestinal and serum level when compared to M_PI_-H mice. These differences were seen at baseline, even without a secondary insult and demonstrated that the gut microbiota not only has local intestinal effects, but also has systemic effects including inducing a systemic inflammatory response and influencing overall growth.

Poor early postnatal growth after preterm birth is clinically associated with short term morbidities in the neonatal intensive care unit as well as long-term adverse neurological outcomes. Although the mechanism is not fully understood, contributing independent factors include severity of illness and nutritional delivery^18–20^. We hypothesized that early microbiota development also influences brain development contributing to long term neurodevelopmental potential. Specifically, we postulated that microbiota associated with impaired growth phenotypes adversely influence brain development. We thus investigated several potential mechanisms including growth factors, neuroinflammation, brain fatty acid compositions, and microbial metabolites by which microbiota may influence both growth and neurodevelopment.

Deficits in growth hormones such as circulating insulin-like growth factor 1(IGF-1) contribute to adverse outcomes in preterm infants including poor postnatal growth^21–24^. Patients with mutations in the *igf-1* gene or in the *igf1r* gene have severe somatic growth failure, microcephaly, and severe cognitive impairment^25^. Specifically in preterm infants, IGF-1 circulating levels are positively correlated with birth weight, length, and head circumference^26^. Furthermore, circulating IGF-1 has specifically been shown to be an important regulator of brain cell proliferation, apoptosis, myelination, neurogenesis, maturation and differentiation^27^. Studies in GF mice have reported that microbiota-regulated IGF-1 levels in mice drive juvenile growth^28^. Since IGF-1 regulates brain development and microbiota-mediated IGF-1 has been shown to regulate growth, we hypothesized that the two growth phenotype associated-microbiomes could regulate both somatic growth and brain development through IGF-1.

Preterm infants are at high risk for neuroinflammation resulting from exposure to maternal, fetal, and neonatal infection and non-infectious insults^29^. Neuroinflammation can give rise to common neonatal brain injuries such as periventricular leukomalacia (PVL) and hypoxic-ischemic encephalopathy^30^. These neuroinflammation-related brain injuries are associated with neurological disorders such cerebral palsy, autism and schizophrenia^31,32^ and can contribute to long term adverse neurological outcomes such as motor and cognitive deficits^33,34^. Gut microbial cell wall components continually interact with the innate immune system to induce the secretion of cytokines. In our previous study^15^, pro-inflammatory mediators such as IL-1β, TNF, and IFNγ were elevated in the serum of mice colonized with microbes from the poor growth infant, reflecting a systemic effect by the microbiome. However, the effect of the microbiota on neuroinflammation such as IL-1β and TNF was not investigated at the time.

Long-chain polyunsaturated fatty acids (PUFA) such as arachidonic acid (AA) and docosahexaenoic acid (DHA) are essential structural and functional constituents of cell membranes and are required for the growth and function of the brain and vascular systems^35^. Brain accumulation of monounsaturated fatty acids such as oleic acid (C18:1) and tetracosenoic acid (24:1), which is elongated from C18:1 ω-9, is very high during the early postnatal period when myelin is being formed most rapidly^36^. The incorporation of these fatty acids in the fetal brain occurs mainly during the last trimester of pregnancy and continues to the end of two years of life^37,38^ thus preterm infants are at high risk for PUFA deficiency and this deficit-related impaired visual and cognitive functions^39–42^.

Other trajectories of microbiota-originated communication with the brain might involve their metabolites such as short chain fatty acids (SCFAs) generated by enteric bacterial fermentation^43^. SCFAs have been implicated in the pathogenesis of several neurodegenerative diseases^44^, with perhaps the strongest evidence that SCFAs have roles in the gut-brain axis coming from a study demonstrating that SCFA-producing bacteria or butyrate itself can decrease the blood-brain barrier (BBB) permeability in GF mice^45^.

The microbiome has increasingly become a focus of studies of host development and functions^46–48^. However, its impact on early postnatal brain development remains elusive. The cause of poor neurodevelopmental outcome in preterm infants is certainly multifactorial; however a key distinctive of the microbiome is that it is modifiable for an individual infant in a time frame parallel to brain development. In this study, we introduced two growth phenotype-associated microbiotas to pregnant GF mice and investigated the influence of these two distinct microbial communities on the postnatal brain development of the offspring. We further investigated multiple potential mechanisms by which microbiota can affect brain development and demonstrated that the microbiota’s effects may be partially mediated by circulating IGF-1 levels and markers of neuroinflammation.

## Results

### Microbiota influences neuronal development

**Figure 1:**
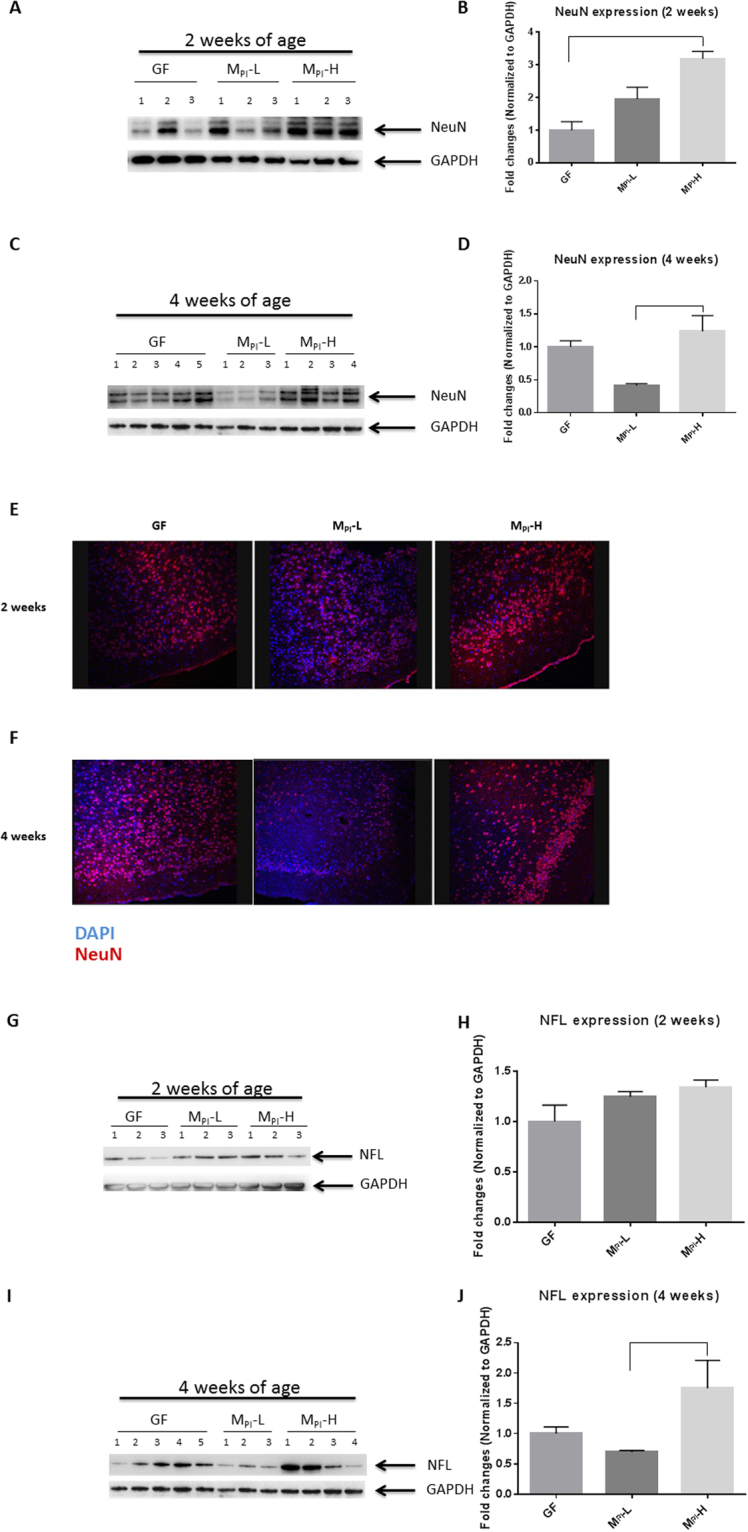
Regulation of neuronal development by gut microbiota. Neuron development was evaluated by western blot and immunohistochemistry. (**A, B**) Representative images and quantification of western blots of the expression of NeuN at two weeks of age in GF, M_PI_-L and M_PI_-H mice (all n = 3). (**C, D**) Representative images and quantification of western blots of the expression of NeuN at four weeks of age in GF (n = 5), M_PI_-L (n = 3) and M_PI_-H mice (n = 4). (**E**, **F**) Representative images of immunofluorescence labeling of NeuN (Red) specific for neurons, counterstaining with DAPI (blue) to visualize all cells, showing decreased NeuN in mouse cortex sections in the M_PI_-L pups compared to M_PI_-H mice at both two and four weeks of age. (**G**, **H, I, J**) Representative images and quantification of western blots showing the expression of NFL at two and four weeks of age, respectively (for two weeks n as indicated for previous data; for four weeks GF (n = 7), M_PI_-L (n = 5) and M_PI_-H mice (n = 4)). One-way ANOVA was used to detect the difference among groups. Bars with indicates a significant difference between the two bars (at least *p* < 0.05). Blots were cropped and the original blots are presented in Supplementary Figure S1.

One of the hallmarks of brain development is neuronal differentiation with permanent exit from the mitotic cycle. NeuN is a 46/48-kD nuclear protein antigen used widely to identify postmitotic mature neurons in both research and diagnostics^49^. Increased expression of neurofilament proteins (NFs), which provide support for axonal growth, is closely associated with continued growth of axons and axon diameter^50^. To examine whether microbial colonization impacts early neuronal development, we measured the expression levels of NeuN and NF, markers of early development in the brain. In our study, we colonized pregnant GF mice at E15 with the human microbiome of interest, and the newborn pups were not separated from the colonized mother until weaning thus the offspring acquired a microbiota reflecting the human microbiome of interest from contact with the mother^15^. The fecal samples were from the first two postnatal weeks in human preterm infants with two different growth rates (M_PI_-H and M_PI_-L) during their NICU course, and resulted in corresponding high and low growth phenotypes in the pup offspring^15^. In this study, western blot analysis of cerebral cortex homogenates with anti-NeuN antibody showed significantly increased levels of NeuN expression in M_PI_-H mice colonized with microbiota from a preterm infant with good growth compared with GF pups at two weeks of age (Fig. 1A,B). M_PI_-L pups colonized with microbiota from a preterm donor with poor growth demonstrated significantly lower NeuN expression compared to M_PI_-H mice colonized with a preterm donor with a good growth at four weeks of age (Fig. 1C,D). These observations were further demonstrated by immunostaining (Fig. 1E,F) at both two and four weeks of age.

There was no significant difference in NFL expression among the experimental mice at two weeks of age (Fig. 1G,H). At four weeks of age, M_PI_-L mice had significantly lower expression of NFL when compared to M_PI_-H mice (Fig. 1I,J). Taken together, these data demonstrate a microbiota-dependent delay of neuronal development in the brains of M_PI_-L mice compared to M_PI_-H mice.

### Microbiota influences oligodendrocyte development

**Figure 2:**
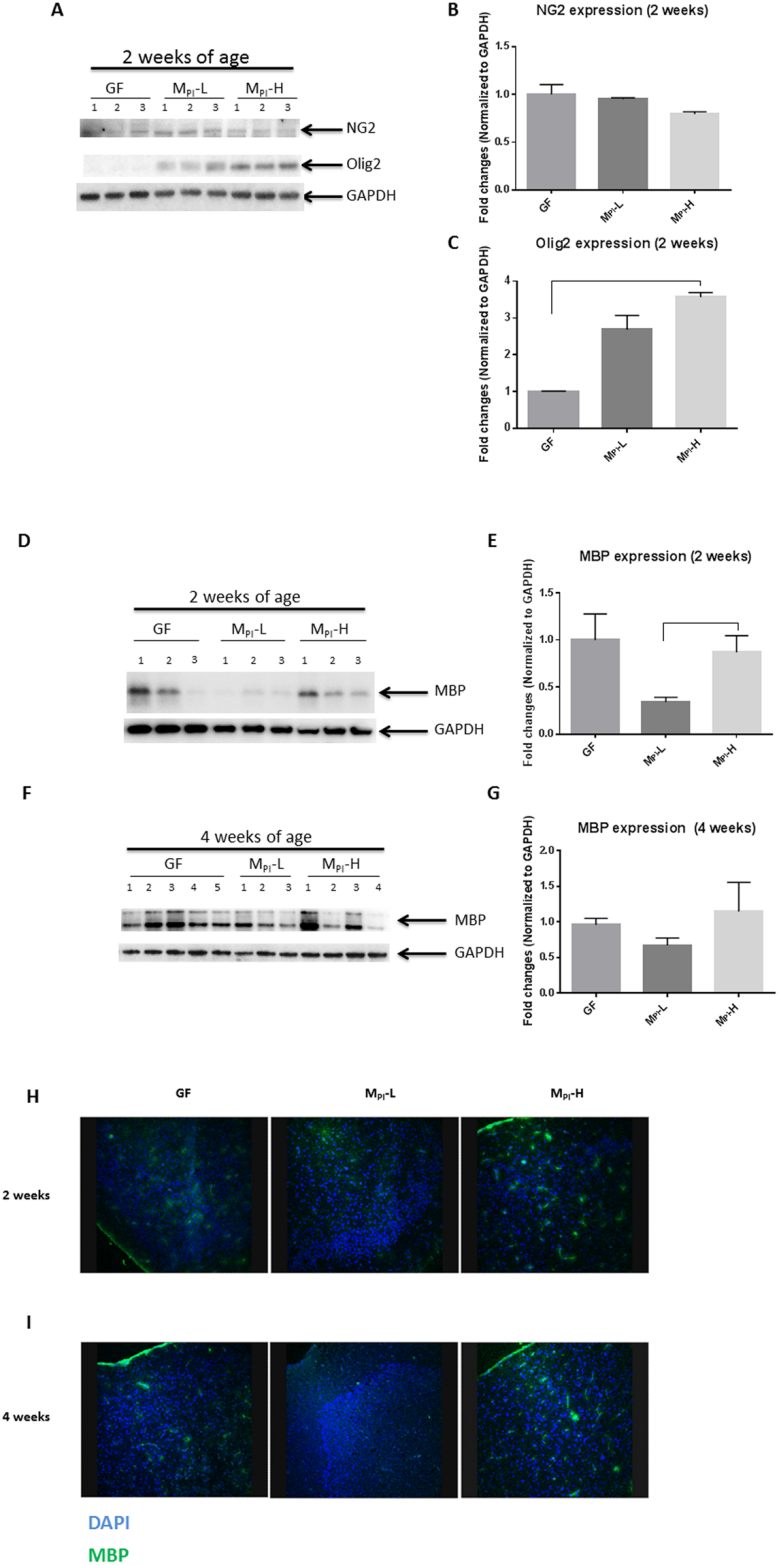
Regulation of cortex myelination by gut microbiota. Development of oligodendrocytes was evaluated by western blot and immunohistochemistry. (**A, B, C**) Representative images and quantification of western blots showing the expression of NG2, an oligodendrocyte progenitor cell marker, and Olig2, a marker for pre-myelinating oligodendrocytes at two weeks of age in GF, M_PI_-L and M_PI_-H mice (all n = 3). Representative images and quantification of western blots showing the expression of MBP (**D, E**) at two weeks of age in GF (n = 5), M_PI_-L (n = 5) and M_PI_-H mice (n = 5) and (**F, G**) at four weeks of age in GF (n = 7), M_PI_-L (n = 5) and M_PI_-H (n = 4) mice. (**H**, **I**) Representative images of immunostaining of MBP (green), counterstaining by DAPI (blue) for nuclei, showing the decreased MBP in mouse cortex sections in the M_PI_-L pups compared with M_PI_-H mice at both two and four weeks of age. One-way ANOVA was used to detect the difference among groups. Bars with indicates a significant difference between the two bars (at least *p* < 0.05). Blots were cropped and the original blots are presented in Supplementary Figure S2.

Nerve/glial-antigen 2 (NG2) is expressed by oligodendrocyte precursor cells (OPC) but not by any other mature neural cell-type^51^. OPCs under defined regulation first differentiate into premyelinating oligodendrocytes (OLs) where OL-specific transcription factors such as Olig2 drive the transcription of genes required for differentiation^52^. Differentiated OLs myelinate axons of neurons to ensure rapid propagation of action potentials and provide metabolic support for axons. Myelin basic protein (MBP) is the major structural element of myelin and is essential for axon myelination, compacting, and wrapping and is developmentally regulated^53^. Protein expression levels tested by western blot demonstrated that cerebral cortex expression of NG2 was not statistically different among the three experimental groups at two weeks of age (Fig. 2A,B). Expression of Olig2 was higher in M_PI_-H compared to GF mice at 2 weeks of age (Fig. 2A,C). NG2 and Olig2 were only minimally detected at four weeks of age in all groups (data not shown).

Immunoblot analysis of cerebral cortex samples from two-week old mice revealed that M_PI_-L demonstrated significantly reduced MBP expression when compared to M_PI_-H mice (Fig. 2D,E). MBP expression at four weeks of age was not different among these groups (Fig. 2F,G). These results were further demonstrated by immunostaining as shown in Fig. 2H,I. These data demonstrate that microbiota from a poor growth preterm infant is associated with delayed oligodendrocyte development and myelination in the early (two weeks of age) postnatal brain.

### Microbiota influences neurotransmission pathways

**Figure 3:**
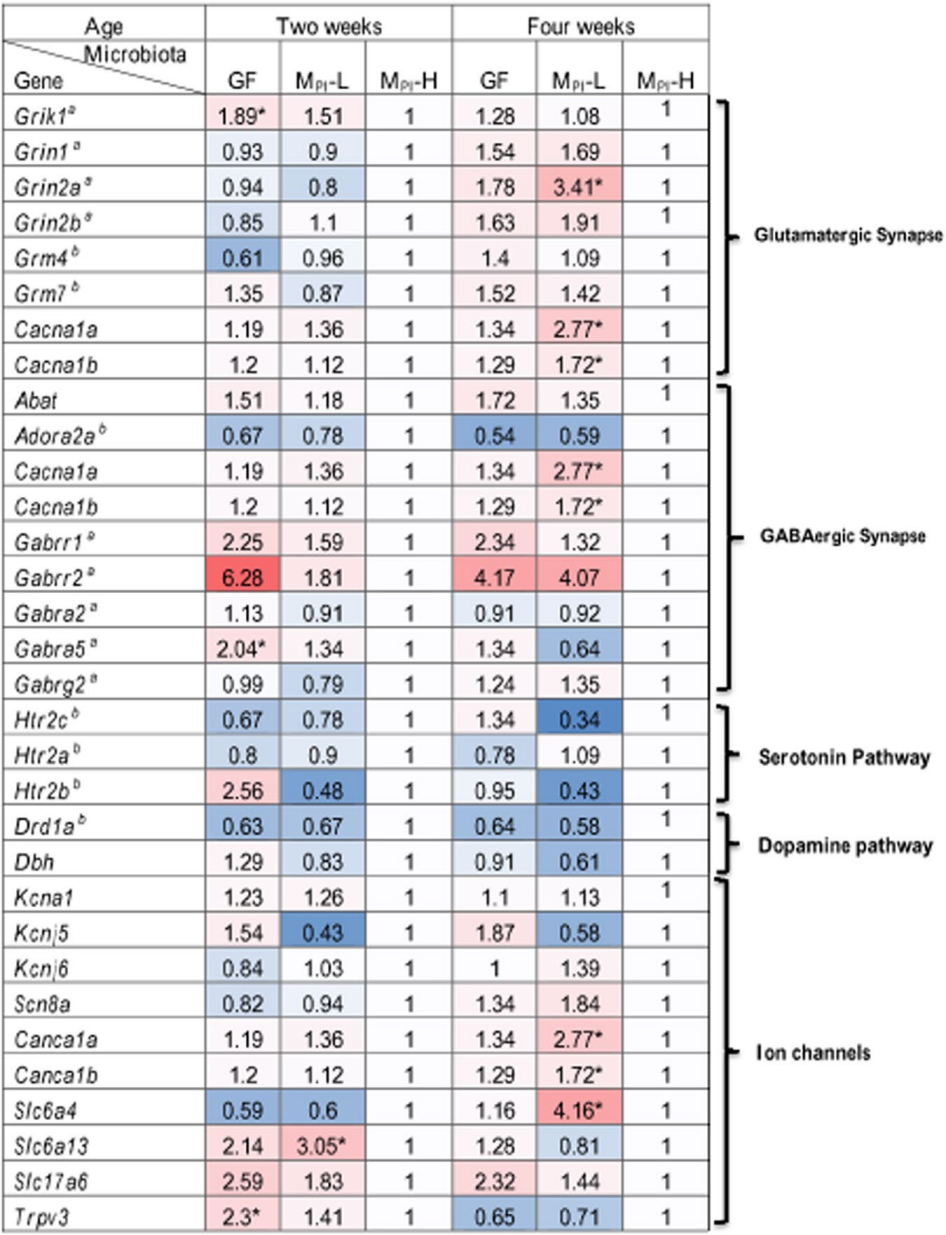
Effect of microbiota on gene expression related to neurotransmission pathways. Expression of genes related to neurotransmission pathways. ^a^Represents ionotropic receptors and ^b^represents metabotropic receptors. Transcript values measured by RT-PCR were first normalized to *Gapdh* (n = 3–9 for two weeks and n = 3–7 for four weeks). Comparison to M_PI_-H values as fold changes are presented in heatmap format to highlight differential effects on gene expression in GF, M_PI_-L and M_PI_-H mice. *Indicates a statistical difference when compared to M_PI_-H mice. In this pseudo-colored heat map, increasing red intensities indicate genes with higher expression levels compared to M_PI_-H mice, and increasing blue intensities indicate genes with lower expression levels compared to M_PI_-H mice.

Glutamate and *γ-*aminobutyric acid (GABA) are the main excitatory and inhibitory neurotransmitters in the central nervous system, respectively. Together they are involved in over 85% of the synapses that underlie learning and memory, motor activity, sensory and many other functions in the mammalian cortex^54–56^. Serotonergic and dopamine signaling control mood, sleep, concentration, and motivation. Dysfunction and imbalance of these pathways can contribute to cognitive deficits^57,58^. It has been suggested that changes in serotonergic signaling may contribute to the altered anxiety phenotype in GF mice^59^. Since previous molecular and behavioral studies have implicated the gut microbiota in the development of neuronal circuits^9,60^, we studied the expression of synaptic transmission plasticity-related genes by quantitative RT-PCR (Fig. 3). Microbiota indeed impacted Glutamatergic, GABAergic, serotonergic, dopaminergic and ion channel pathways. Furthermore M_PI_-L and M_PI_-H had differential impacts on different neurotransmission pathways. For example, at four weeks of age, M_PI_-L was generally associated with increased glutamatergic pathway activity demonstrated by increased expression levels (red) and decreased serotonin and dopamine pathway expression levels (blue) in Fig. 3 when compared to M_PI_-H.

### Colonization of GF mice with human fecal samples from a preterm infant with poor growth is associated with neuroinflammation in the brain

**Figure 4:**
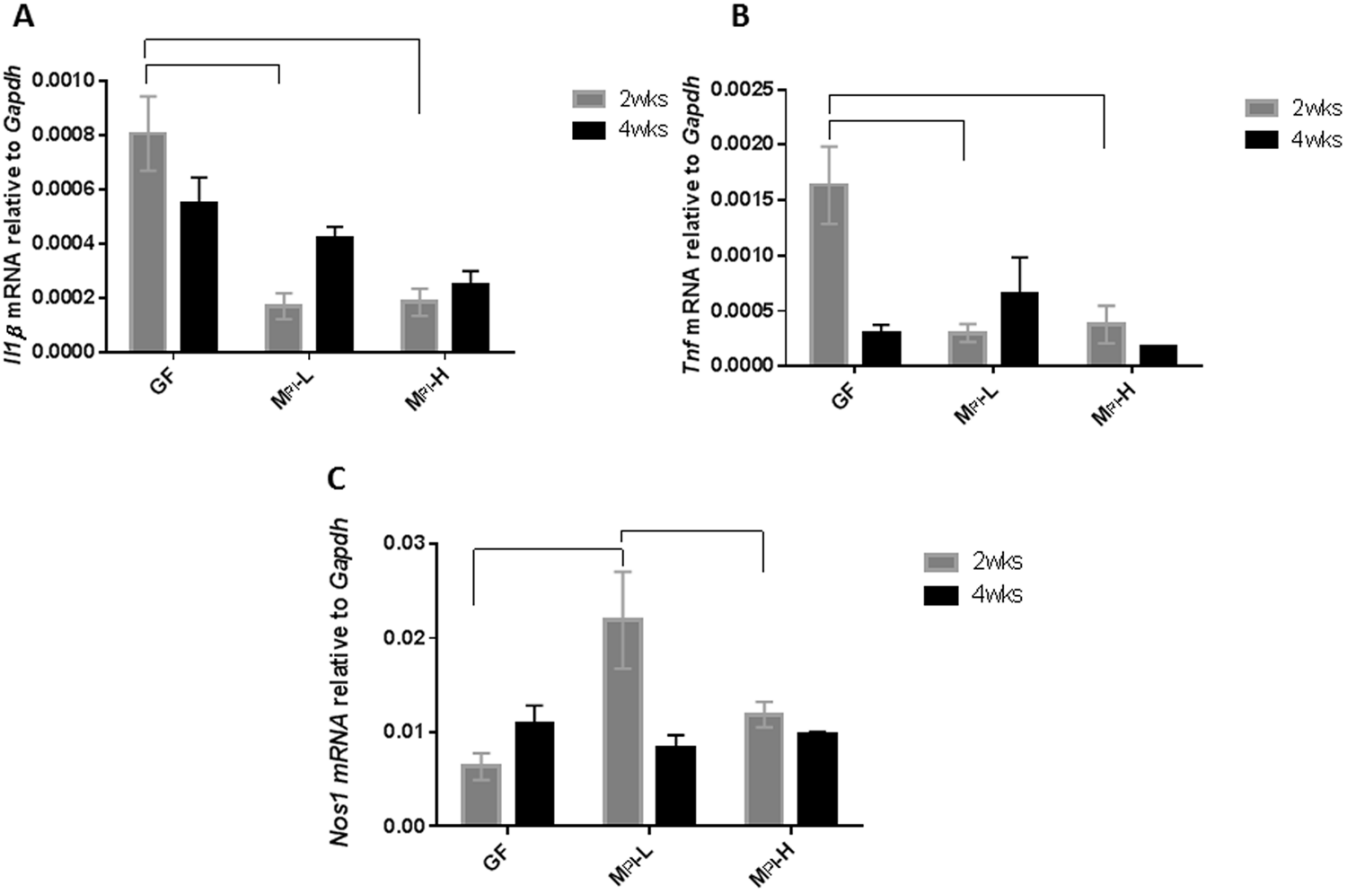
Effects of microbiota on neuroinflammation. Relative brain transcripts of *Il-1β* (**A**), n = 3–9 at two weeks and n = 2–8 at four weeks of age), *Tnf* (**B**), n = 3–9 at two weeks and n = 2–6 at four weeks of age), and *Nos1* (**C**), n = 3–8 at two weeks and n = 2–7 at four weeks of age) were measured by RT-PCR. Two-way ANOVA was used to detect the effects of age and microbiota among the groups. Post-*hoc* test was used to identify differences when a main effect was noted. Bars with  indicates a significant difference between the two bars (at least *p* < 0.05).

IL-1β and TNF are major regulators of neuroinflammation associated with an inflammatory/cytotoxic phenotype in the brain^61,62^. Neuronal nitric oxide synthase (NOS1) is a key enzyme implicated in neurotoxicity in the perinatal cortex^63,64^. As a potential mechanism by which different microbiota can affect brain development, we examined the overt inflammatory status in the developing brain. Our data showed that at two weeks of age, GF mice had significantly higher *Il-1β* and *Tnf* mRNA expression in the brain when compared to M_PI_-L and M_PI_-H mice (Fig. 4A,B). M_PI_-L mice exhibited significantly higher *Nos1* expression when compared to GF and M_PI_-H mice in the cortex (Fig. 4C). These data demonstrate that microbiota can affect the neuroinflammation status of the developing brain.

### Colonization of GF mice with human fecal samples from preterm infants did not change the brain fatty acids profile

**Figure 5:**
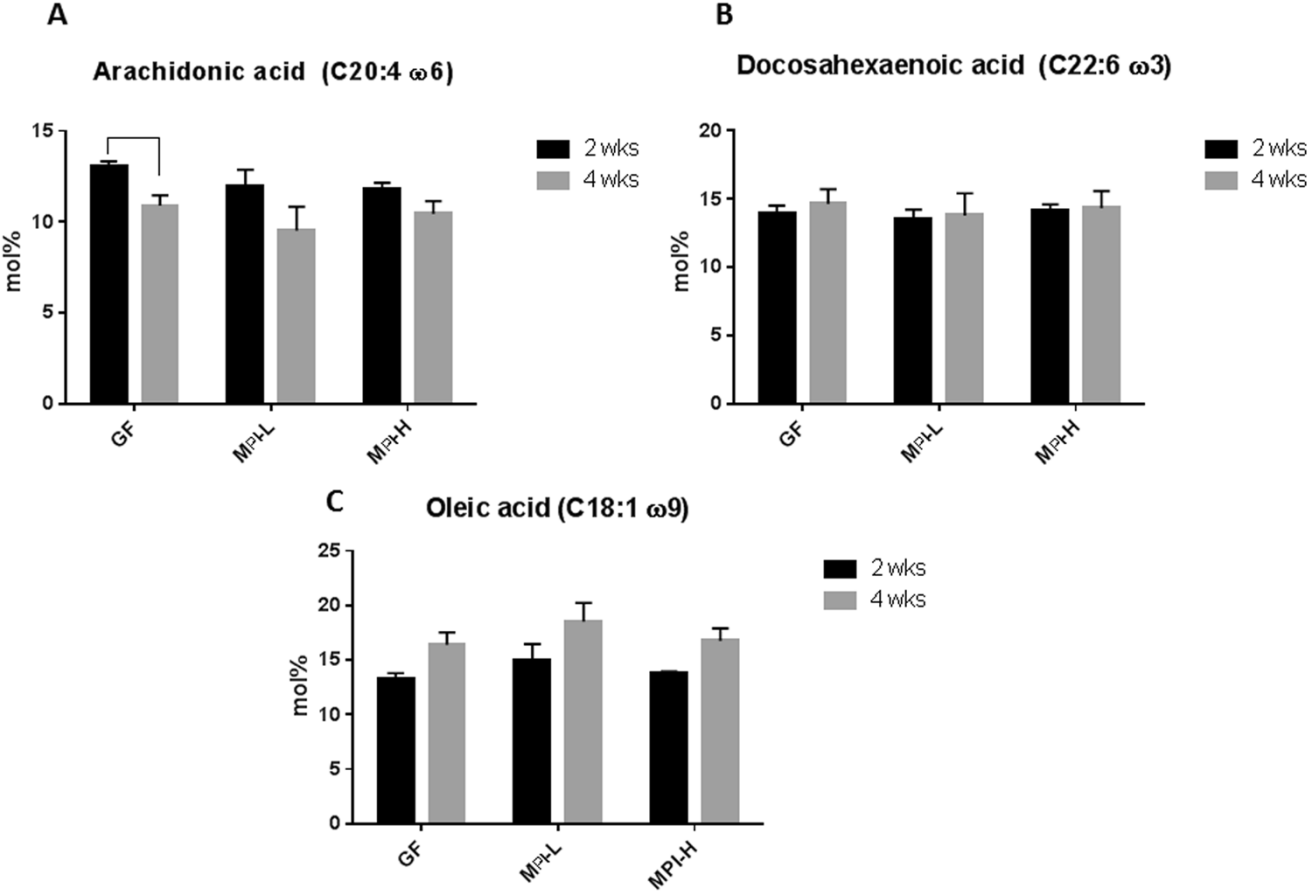
Effects of microbiota on brain fatty acid profile. Brain AA (**A**), DHA (**B**), and oleic acid (**C**) contents were measured by GC in the brain tissues collected both at two (n = 3–8) and four (n = 3–6) weeks of age. Two-way ANOVA was used to detect the effects of age and microbiota among the groups. Data are presented as mol% ± s.e.m.

Preterm infants are subjected to brain fatty acid deficiency because fatty acid accumulation in the brain is at the highest rate from the intrauterine and neonatal period up to two years of age^37^. To evaluate if there were effects of growth phenotype–related preterm infant microbiota on brain fatty acid composition, we measured several PUFAs and myelin-related fatty acid levels in the brain. The levels of C20:4 (AA) (Fig. 5A), C22:6 (DHA) (Fig. 5B) and C18:1 (Oleic acid) (Fig. 5C) at both two weeks and four weeks of age were not different among GF, M_PI_-L and M_PI_-H mice, demonstrating the colonization of microbiota from either a poor growth preterm infant or a good growth preterm infant did not influence these fatty acids in the brain.

### Effects of different microbiota colonization to GF mice on fecal SCFA concentration

**Figure 6:**
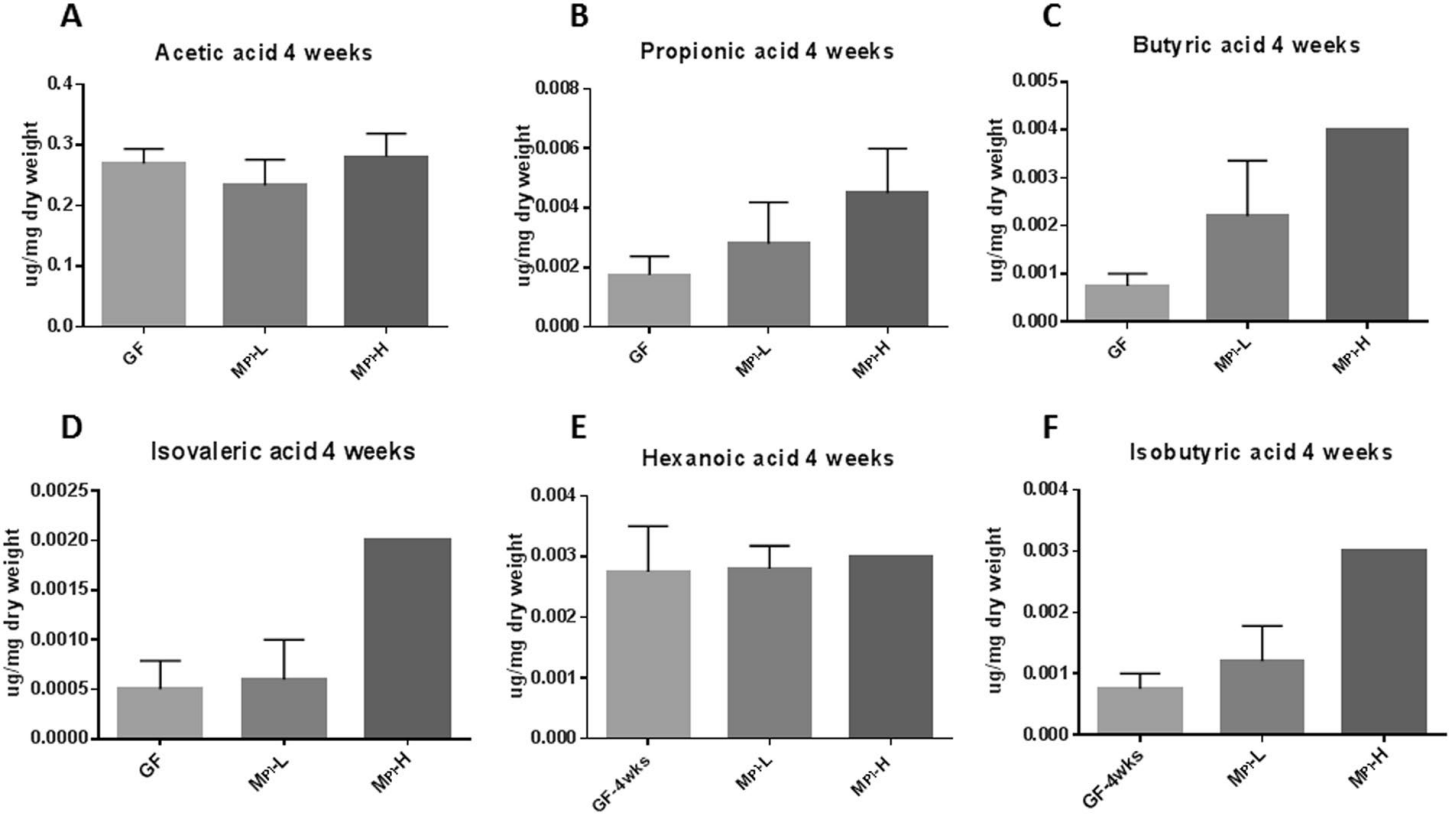
SCFA analysis of fecal samples. Effects of microbiota on fecal acetic acid, propionic acid, and butyric acid at four weeks of age were measured by GC. Fatty acid composition is expressed as a percent of total identified fatty acids and concentrations as µg/mg sample. One-way ANOVA was used to detect differences among groups.

SCFAs are among the major metabolites produced by anaerobic bacterial fermentation in the gut^65^. We found that fecal contents of acetic acid (Fig. 6A), propionic acid (Fig. 6B), butyric acid (Fig. 6C), isovaleric acid (Fig. 6D), hexanoic acid (Fig. 6E), and isobutyric acid (Fig. 6F) were not different among the three groups at four weeks of age, demonstrating the colonization of microbiota from either a poor growth preterm infant or a good growth preterm infant did not influence the SCFA production in the fecal samples in our experimental setting.

### Colonization of germ free mice with human fecal samples from a preterm infant with poor growth resulted in decreased circulating IGF-1, IGFBP3 levels and brain IGF-1 levels and increased *Igfbp3* brain transcript levels

**Figure 7:**
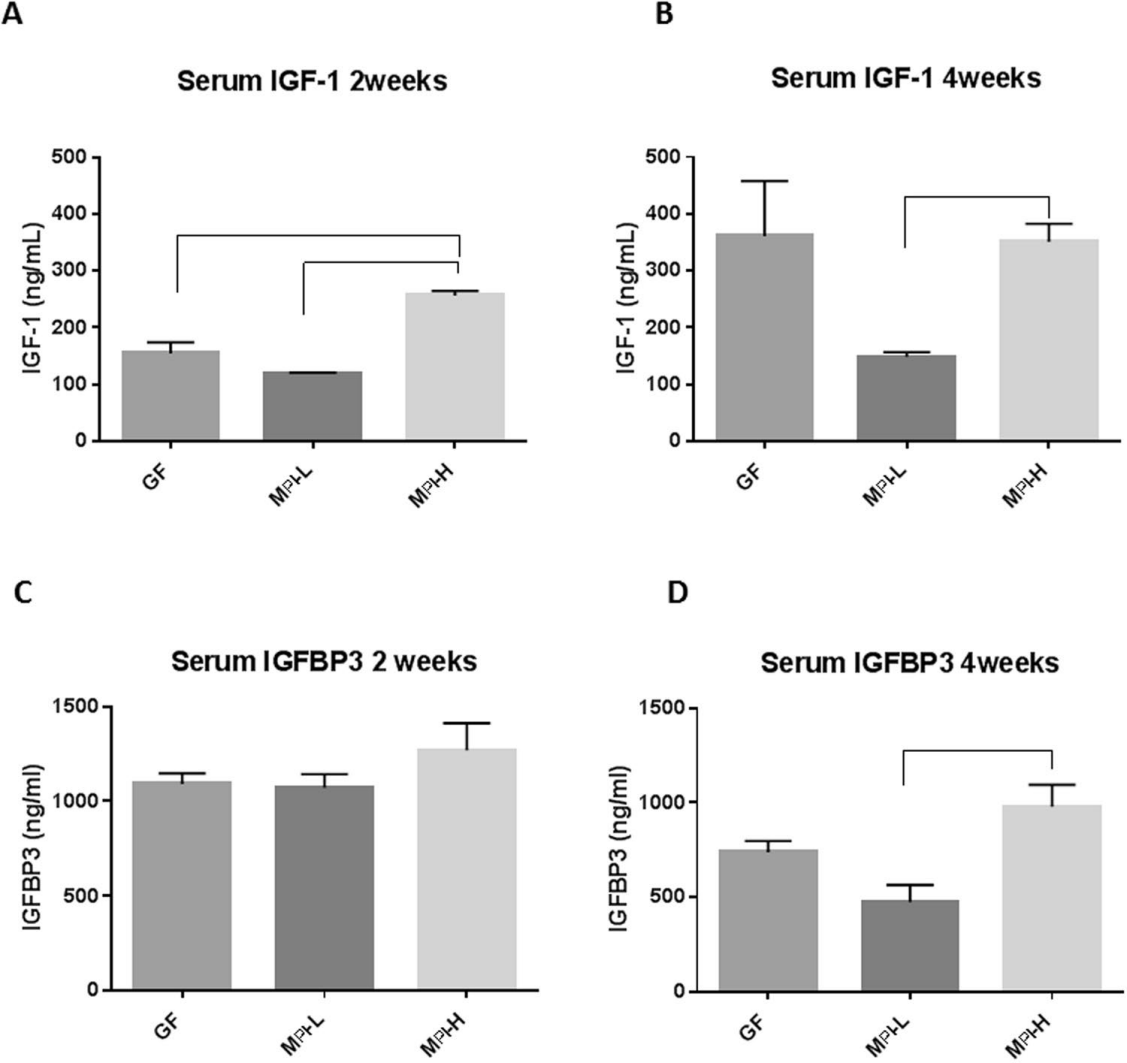
Effects of microbiota on the levels of circulating IGF-1 and IGFBP3. Circulating IGF-1 (**A**,**B**) and IGFBP3 levels (**C**,**D**) were measured by ELISA at both two weeks (n = 3–6) and four weeks (n = 3–8) of age. One-way ANOVA was used to detect the difference among the groups. Bars with  indicate a significant difference between the two bars (at least *p* < 0.05).

To understand the mechanisms by which different growth phenotype related-microbiota colonization resulted in different brain development phenotypes, we tested whether the effect of microbiota is mediated through circulating levels of IGF-1. Serum levels of IGF-1 measured by ELISA were significantly lower in GF and M_PI_-L mice compared with the M_PI_-H mice at two weeks of age (Fig. 7A, *p* < 0.05). At four weeks of age, M_PI_-L mice still had significantly lower circulating IGF-1 compared to M_PI_-H mice (Fig. 7B, *p* < 0.05). In serum, the majority of the IGFs exist in a 150-kDa complex including the IGF molecule, IGF binding protein 3 (IGFBP-3), and the acid labile subunit (ALS). This complex prolongs the half-life of serum IGFs and facilitates their endocrine actions. We found that there was no difference in serum IGFBP3 levels among the mice at two weeks of age (Fig. 7C), but at four weeks of age, IGFBP3 serum level in M_PI_-L mice was significantly lower than that of the M_PI_-H mice (Fig. 7D, *p* < 0.05). These data suggest that poor growth-phenotype related microbiota is associated with reduced circulating IGF-1 and IGFBP3 levels in mouse pups.

**Figure 8:**
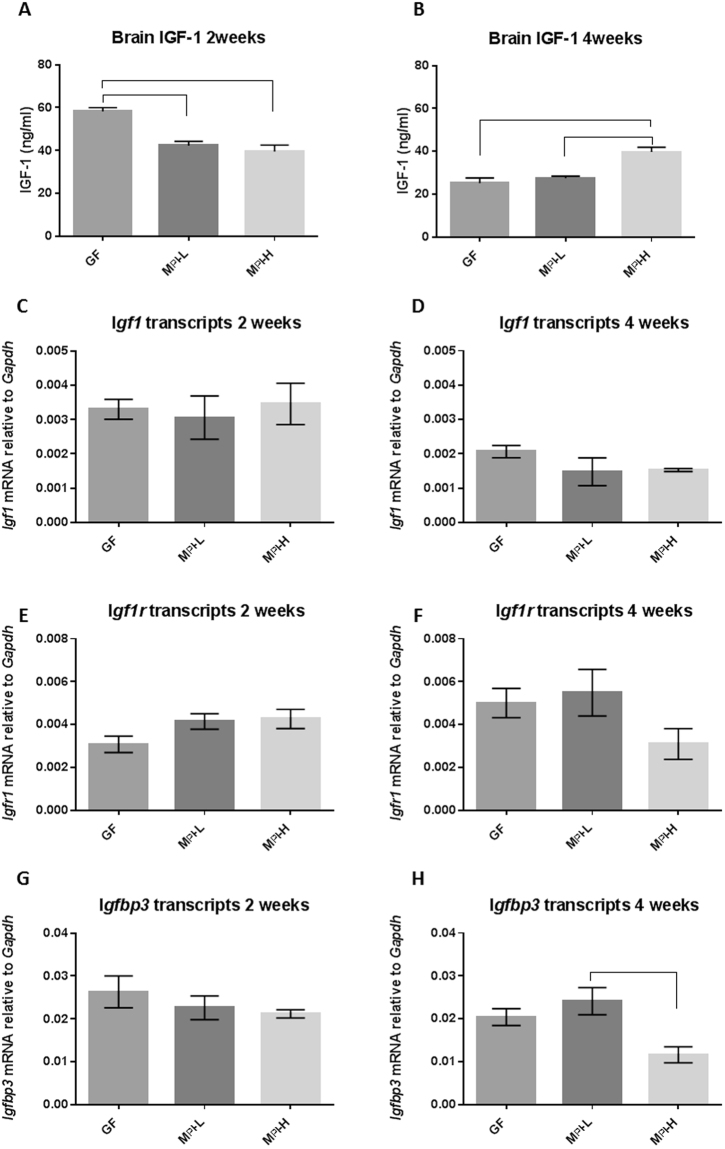
Effects of microbiota on the brain levels of IGF-1, IGF1R and IGFBP3. Brain IGF-1 (**A**,**B**) were measured by ELISA at both two weeks (n = 3–7) and four weeks (n = 2–6) of age. Relative brain transcripts of *Igf1* (**C**, n = 2–9 for two weeks and **D**, n = 2–7 for four weeks), *Igf1r* (**E**, 2 weeks) and (**F**, four weeks), and *Igfbp3* (**G**, two weeks) and (**H**, 4 weeks) (all n = 3–9 for two weeks and n = 2–8 for four weeks) were measured by RT-PCR. One-way ANOVA was used to detect the difference among the groups. Bars with  indicates a significant difference between the two bars (at least *p* < 0.05).

To assess contribution of circulating IGF-1 to brain IGF-1 status, we measured local production of IGF-1 in the brain at both the protein and mRNA level. The brain IGF-1 levels of GF mice were significantly higher than those of M_PI_-L and M_PI_-H pups (at least *p* < 0.05) at two weeks of age (Fig. 8A). At four weeks old, the levels of IGF1 in the brains of both GF and M_PI_-L mice were significantly lower than those of M_PI_-H mice (Fig. 8B). However, there were no differences in brain *Igf1* (Fig. 8C,D) or *Igf1r* (Fig. 8E,F) mRNA levels among the three experimental groups at either two weeks or four weeks old of age.

IGFBP3 antagonizes the local biologic effects of IGF-1 by having higher affinity for IGF-1 than the IGF-1 receptor^66,67^. At two weeks of age, there were no differences in *Igfbp3* transcript levels among the three experimental groups (Fig. 8G). At four weeks old of age, M_PI_-L mice had significantly higher *Igfbp3* mRNA levels than M_PI_-H mice (Fig. 8H, *p* < 0.05). These data demonstrate that poor growth phenotype-associated microbiota resulted in decreased circulating and brain IGF-1, decreased circulating IGFBP3 and increased IGFBP3 brain levels when compared to good growth phenotype-associated microbiota.

### Colonization of GF mice with human fecal samples from a preterm infant with poor growth decreased liver IGF-1 levels

**Figure 9:**
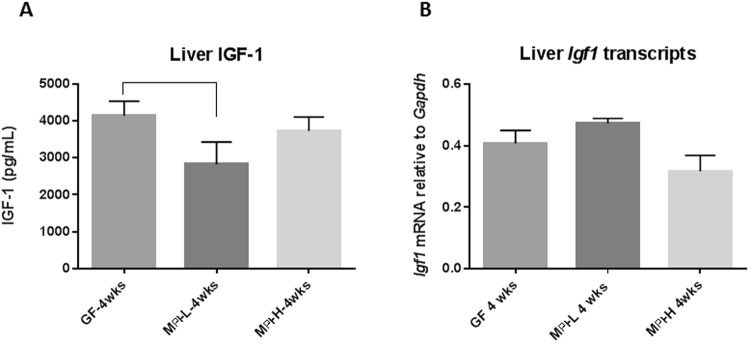
Effects of microbiota on the liver protein and mRNA levels of IGF-1. Liver IGF-1 (**A** was measured by ELISA at four weeks n = 2–5) of age. Relative liver transcripts of *Igf1* (**B**, n = 2–5) at four weeks of age was measured by RT-PCR. One-way ANOVA was used to detect the difference among the groups. Bars with  indicate a significant difference between the two bars (at least *p* < 0.05).

Liver synthesis and secretion of IGF-1 is responsible for 80% of the circulating IGF-1. To test whether the effect of different microbiota on circulating IGF-1 level is due to liver production of IGF-1, we examined both the protein and transcript levels of IGF-1 in the liver. IGF-1 tissue levels measured from liver homogenates by ELISA were significantly lower in M_PI_-L mice compared to GF mice (Fig. 9A, *p* < 0.05) at four weeks of age. Liver *Igf1* transcript levels were not affected by the different microbiota (Fig. 9B). Our data demonstrate that microbiota from a preterm infant with poor growth negatively regulated liver levels of IGF-1.

## Discussion

Growth velocities in the lowest quartile of extremely preterm infants while they are in the neonatal intensive care unit are associated with an increased risk of long term neurodevelopmental impairment^68^. Using a humanized mouse model of microbiome-induced differential growth phenotypes^15^, we demonstrate that the intestinal microbiota is an important mediator in this growth-neurodevelopment relationship with an impact on IGF-1, brain development, and neuroinflammation. Together with our previous study^15^, our body of work demonstrates that early infant intestinal microbiota may influence both growth and brain development phenotypes.

In our study, we colonized pregnant GF mice at E15 with specific growth-phenotype associated microbial communities so that pups would be naturally colonized with these distinct microbial communities. GF mice colonized with microbiota from a poor growth human infant exhibited decreased NeuN and NFL at four weeks of age and reduced MBP expression in the cortex at two weeks of age when compared to mice colonized with microbiota from a good growth human infant, suggesting an altered neuronal development and myelination process. Microbiota did not change the brain fatty acid composition and fecal SCFA production, but had diverse impact on neurotransmission pathways. Furthermore, the mechanisms by which microbiota can alter brain development and functions might be due to an impact on circulating level of IGF-1and local inflammation. In our study, microbiota from an infant with poor postnatal growth is associated with lower levels of circulating and brain levels of IGF-1 and higher levels of neuroinflammation markers.

Our study extends the growing body of research to support the view that normal gut microbiota plays a critical role in shaping brain functions^9,69–71^. Although previous studies have provided a strong foundation for establishing the gut-brain axis concept by modulating gut microbiota in adult animals^9,72^, few studies have demonstrated the connection between gut microbiota and neonatal brain development^59^. Interestingly, in one of the earliest studies demonstrating the gut-brain connection, Sudo *et al*.^59^ emphasized that normalization of hypothalamic-pituitary-adrenal response to stress in GF mice existed only when GF mice were reconstituted with SPF feces at one week old but not at three weeks of age. These observations suggest that early establishment of the microbial community might be critical in shaping the brain function and have long-lasting effects on behaviors. Recently, the first study to demonstrate associations between the gut microbiota and cognition in human infants has been published^73^. In the study, Carlson *et al*. demonstrated that fecal microbial community diversity from one year old infants was associated with variation on the Mullen score, visual reception scale, and expressive language scale at two years of age. It has been suggested that there is an early parallel window of microbiome and brain development^10^. Microbiome colonization begins around birth and continues to develop into a relative stable community during the first 2–3 years of life^10^. Critical brain development such as increases in cortical thickness and surface area mostly by neurogenesis occurs faster during the first year than in the second year of life^74^. Axon diameters and myelin sheaths undergo rapid growth as a reflection of white matter maturation during the first two years of life^75,76^. Our study characterized the effect of the early microbiome, from less than two weeks of life in preterm infants, on neuronal and myelination development in the early postnatal ages (pre-weaned and post weaned time points in mice which corresponds to time points in the first year of human life), providing direct evidence that there is a critical window in early life during which microbiota can influence brain development.

Other impacts of microbiota on brain development observed in this study includes the complex and diverse alterations in gene expression in multiple neurotransmission pathways, including glutamatergic, GABAergic, serotonin and dopamine pathways as well as neurotransmitter transporters and ion channels. Even though the mechanisms by which microbiota affect these pathways are beyond the scope of this study, these findings provide evidence that microbiota can have diverse impacts on the brain. For instance, developmental changes in NMDA receptor composition at early postnatal ages may influence the balance of plasticity and stability that is critical for information processing and storage and precedes the associative learning^77^. Thus our data also suggests that early microbiota colonization associated with a poor growth outcome might pose neurological disadvantages for neonates by affecting neurotransmission pathways.

Extremely preterm infants exposed to a prolonged systemic inflammatory response are at an increased risk for impaired neurodevelopment^78,79^. A persistent systemic inflammatory response during a sensitive period of postnatal life in a hospital setting influences critical phases of myelination and cortical plasticity^29,30^. Our previous study demonstrated a systemic proinflammatory profile in mice colonized with microbiota from a preterm infant with poor growth^15^. Elevated proinflammatory cytokines have been associated with brain injuries in the developing neonatal brain^80,81^. However, even though phenotypic changes in brain development have been linked to systemic changes of proinflammatory cytokines, the underlying mechanisms that transfer systemic inflammation to the brain remains elusive^29^. Possible mechanisms by which peripheral cytokines might influence brain inflammation include the immature development of the BBB^82^ and interaction between TLR ligands and brain endothelial TLR receptors followed by activation of the downstream signaling to induce inflammation in the brain^83^. Perinatal neuroinflammation has been associated with neurotoxicity^63^ and dysfunctions of synaptic organization in the developing brain^29^. In animal models of neonatal meningitis, cerebral cortex and hippocampal expression of TNF, IL-1β and IL-6 were upregulated after *S*. *Pneumonie* or *S*. *agalactiae* infection in the neonatal rats^84^. We demonstrate that, even without induced active infection, different baseline microbiota colonization patterns result in distinct inflammatory profiles in the brain as evidenced by the elevated expression levels of *Nos1* in M_PI_-L mice and *Il-1β* and *Tnf* in GF mice. Our data suggest that the effects of microbiota on neuroinflammation can be another mechanism by which microbiota affect brain development.

The effect of microbiota on serum IGF-1 levels could also contribute to the altered brain development. IGF-1 has been shown to play prominent roles in CNS development and maturation^85^. Homozygous *Igf1 −/−* mice at two months of age had reduced brain weights, white matter size and spinal cord due to the decreased numbers of axons and oligodendrocytes. Myelinated axons, the volume of the dentate gyrus granule cell layer, and the number of striatal parvalbumin-containing cells were also reduced^86^. Moreover, IGF-1 is very important in prenatal and early postnatal neurogenesis^87^ as increased IGF-1 levels result in increased numbers of total neurons in the cerebral cortex. In this study, we have demonstrated that colonization of microbiota from a human preterm infant with poor growth resulted in reduced serum levels and liver production of IGF-1. However, microbiota did not affect the local brain production of IGF-1 evidenced by the similar *Igf1* transcripts in the brain across groups. Our data suggest that serum IGF-1, not locally produced IGF-1, may be the main contributor to the different brain IGF-1 levels in our three experimental groups. Furthermore, microbiota also affected the brain level of *Igfbp3* mRNA, suggesting that microbiota may influence brain IGF-1 bioavailability.

The effect of microbiota on liver production of IGF-1 may contribute to the decreased IGF-1 circulating levels in M_PI_-L mice since the liver expression levels of *Igf1* in M_PI_-L mice were significantly lower than that of GF mice. Modulation of liver production of IGF-1 by microbiota has been shown in several studies; however the extent of the influence is inconclusive. Schwarzer *et al*. report higher liver *Igf1* transcripts in SPF mice compared with GF mice^28^. Yan *et al*. observed higher liver production of IGF1 in GF mice colonized with adult SPF microbiota but no transcript difference was detected^8^. Our study demonstrates that liver production of IGF-1 at early postnatal ages can be modulated by early microbial colonization. The associated reduced circulating IGF-1 levels contribute to the reduced brain IGF-1 levels in M_PI_-L mice and correlates with the delayed neuronal development and myelination in the cortex in these pups. Thus, early alteration of microbial colonization may modulate systemic IGF-1 levels that in turn might impact postnatal brain development.

In conclusion, this study builds on our previous findings demonstrating that early microbiome colonization patterns influence growth trajectories, and suggests that colonization of different microbial communities influences brain development. The initial colonization of microbiota can therefore be a modifiable factor that affects brain development. Future studies on specific involvement of certain microbial communities and their metabolites in altering brain development and functions in early postnatal age are warranted and of clinical importance. Early optimization of microbial colonization at an early postnatal stage might benefit preterm infants to shape brain development and enhance long-term neurological outcomes.

## Methods

### Subjects

This study was designed to investigate the influence of early microbiota on the brain development using gnotobiotic mice transplanted with early microbiota from preterm infants. Subjects were recruited from the neonatal intensive care unit (NICU) at The Comer Children’s Hospital of the University of Chicago. The procedures were approved by the Institutional Review Board (IRB) at University of Chicago (protocol #14991B) and written informed consent was obtained from all patient parents. All methods in the study on humans were performed in accordance with the guidelines and regulations of the University of Chicago.

### Animals

All animal procedures were approved by the Institutional Animal Care and Use Committee under the animal protocol No. 71703 and performed strictly in accordance with approved Animal Care and Use Protocols (ACUPs) at The University of Chicago. Germ free (GF) C57BL/6 J mice were maintained in the gnotobiotic facility of the Knapp Center for Biomedical Discovery at the University of Chicago and are routinely tested for microbes and parasites by the facility’s staff to ensure germ-free conditions. Colonization experiments were carried out as previously described^15^. Briefly, eight to nine week old pregnant GF mice were transfaunated with fecal samples of the preterm human infant donors on E15. Pups were born naturally and nursed by respective mothers until weaning. Early (<two weeks of life) preterm human infant microbiota as representative of the first microbiota to encounter the host were used in the transfaunation since our previous study showed distinct clustering of microbial samples prior to two weeks of life^16^. Corresponding to the growth phenotype, pups from GF dams transfaunated with fecal samples from an infant with poor growth were identified in this study as M_PRETERM INFANT_-Low (M_PI_-L), and pups from GF dams transfaunated with fecal samples from an infant with good growth were identified as M_PRETERM INFANT_-High (M_PI_-H).

### Western blotting

Brain tissues were homogenized in ice-cold 1× RIPA lysis buffer (50 mM Tris-Cl at pH = 7.5, 150 mM NaCl, 1% NP-40 alternative (EMD Millipore Corporation, La Jolla, CA), 0.5% (wt/vol) Sodium deoxycholate, 0.1% (wt/vol) SDS with protease inhibitors (Roche Diagnostics GmbH, Mannheim, Germany) and spun at 12,000 × g for 10 mins. Tissue lysates were then subjected to Pierce^®^ BCA assays (Thermo Scientific Inc., Waltham, MA) for protein concentration measurement. Equal amount of protein lysate was subjected to SDS-PAGE electrophoresis using Bio-Rad Criterion™ XT 4–12% Bis-Tris precast gels (Bio-Rad Laboratories, Inc., Hercules, CA) and transferred to PVDF membranes using a semi-dry transfer system (Bio-Rad). The membranes were blocked with 5% nonfat milk (Bio-Rad) in Tris-buffered saline (TBS, 0.02 mol/l Tris–HCl, 0.137 mol/l NaCl, pHZ7.5) with 0.1% Tween-20 (TBST) for an hour on a shaker at room temperature. The membranes were cut into pieces depending on the molecular weights of the proteins of interest and then incubated with respective primary antibodies in 5% NFM in TBST overnight at 4 °C. The membranes were washed four times for 10 min each time with TBST and then incubated with secondary antibodies for 1 h at room temperature. The chemiluminescent signal was developed using SuperSignal West Femto maximum sensitivity substrate (Thermo Scientific) and captured using a Molecular Imager^®^ ChemiDoc™ XRS + imaging system (Bio-Rad). The density of each band was quantified with ImageJ (NIH, Bethesda, MD) and normalized to GAPDH and presented as fold change relative to the control.

### Immunohistochemistry

Brains were obtained from mice at postnatal age of two weeks (pre-wean) and four weeks (post-wean) and embedded in OCT. Eight µm sections were cut with a cryostat and the sections were fixed in ice-cold methanol at −20 °C for 20 minutes. After washing with 1 × PBS for three times, the samples were permeabilized with PBS with 0.1% triton (PBST) for 15 mins at room temperature. The samples were then incubated with blocking solution (5% goat serum) in PBST for one hour at room temperature. Before primary antibody application, the tissues were circled using a hydrophobic barrier pen. The tissue sections were incubated with respective 50 µl of primary antibody dilution solution overnight at 4 °C. After wash with PBST for three times for 15 mins, the sections were then incubated with respective fluorophore-conjugated secondary antibodies for one hour at room temperature. The sections were counterstained with DAPI antifade mounting medium (Invitrogen) before coverslips were applied.

### RNA Isolation and Real-time PCR

Total RNA from snap frozen brains and livers were isolated using the RNeasy^®^ Plus Mini Kit (QIAGEN GmbH, Hilden, Germany). 500 ng of isolated total RNA was used to synthesize cDNA using RT^2^ First Strand Kit from QIAGEN. TaqMan probes and primers (Thermo Scientific) were used for gene of interests and the housekeeping gene *Gapdh*. Gene expression was normalized to the housekeeping gene and expressed as fold change of experimental controls.

### Antibodies and reagents

Neuronal nuclei (NeuN), Oligodendrocyte transcription factor 2 (Olig2) and Neural/glial antigen 2 (NG2) antibodies were purchased from EMD Millipore (Billerica, MA). Myelin basic protein (MBP), neurofilament-Light chain (NFL), and GAPDH antibody were purchased from Cell Signaling Technology (Danvers, MA). Secondary anti-mouse-HRP and anti-rabbit-HRP antibodies were purchased from Santa Cruz Biotechnology, Inc. (Dallas, TX).

### ELISA for serum, brain, and liver IGF-1 and IGFBP3

Serum, brain, and liver samples were collected and processed for IGF-1and IGFBP3 measurement using Mouse IGF-I or IGFBP3 Quantikine ELISA Kit from R&D Systems Inc. (Minneapolis, MN) according to the manufacturer’s instructions, respectively. Equal amount of tissue lysates of brain, and liver were applied to ELISA plates after protein concentration measured by Pierce^®^ BCA assays (Thermo Scientific). All ELISAs were read with a Multiskan™ GO Microplate Spectrophotometer (Thermo Scientific) with PathCorrection protocol.

### Brain fatty acid analysis

Brain tissues were collected freshly at two and four weeks of age and snap frozen until analysis. Approximately 50 mg segments of brain were homogenized in 1.0 mL of Phosphate- Buffered Saline solution using a Fisher PowerGen 125 homogenizer. Five hundred microliters of the aliquot was removed and lipids were extracted using a modified Folch method^88,89^. Briefly, after the addition of an internal standard (30 μg of heptadecanoic acid), the sample was mixed with 3 ml of chloroform: methanol (2:1 v/v) and vortexed. The sample was then incubated on ice for 10 min, vortexed and centrifuged at 2500 rpm for 10 min. The bottom infranatant was removed and completely dried under nitrogen gas vapors. The dried sample was then methylated by addition of 0.5 ml 0.0.5 M methanolic NaOH and incubated for 3 min at 100 °C. Once cooled, 0.5 ml of BF3-methanol was added, and the sample was incubated at 100 °C for an additional minute. After cooling, the sample was mixed with 1 ml of hexane, followed by 6.5 mL saturated NaCl solution. The sample was vortexed and centrifuged at 1700 rpm for 4 min and the upper hexane phase was transferred to a fresh vial and quantified by gas chromatography-mass spectroscopy (GC-MS). Fatty acids are expressed as a percent of the total fatty acid mass (mol %).

### Short chain fatty acid (SCFA) analysis

Fecal contents were collected freshly at two and four weeks of age, frozen immediately and stored at −80 °C until analysis. Short chain fatty acids were analyzed at OmegaQuant (OmegaQuant, LLC, Sioux Falls, SD) by gas chromatography (GC) with flame ionization detection. Samples were transferred to a screw-cap glass vial which contained heptanoic acid as an internal standard (C7:0 FFA) (Nu-Chek Prep). De-Ionized Water and 50% Sulfuric Acid Solution (Fisher Scientific, NJ, USA (3.5:1 v/v) was added to each vial. Each sample was homogenized with a homogenizer cleaned between each sample. After homogenization anhydrous ethyl ether (Fisher Scientific) was added to each vial and vortexed for 1 minute. Samples were then centrifuged at 4 °C for 10 minutes at 3800 rpm. An aliquot of the ethyl ether layer was transferred to a GC vial. GC was carried out using a GC2010 Plus Gas Chromatograph equipped with an AOC-5000 autosampler with stack cooler (Shimadzu Corporation, Columbia, MD), and a HP-INNOWAX 30 m column (0.25 mm internal diameter, 0.25 um film thickness; Agilent J&W, USA). The stack cooler was kept below 10 °C while samples were kept on an autosampler. Fatty acids were identified by comparison with a standard mixture of short chain fatty acids (Sigma, St. Louis, MO) which was also used to determine individual fatty acid calibration curves. The 7:0 FFA was used to calculate recovery efficiency of the assay and applied to all fatty acids. Fatty acid composition was expressed as a percent of total identified fatty acids and concentrations as µg/mg sample.

### Statistical analysis

All data are presented as the mean ± s.e.m. One and two-way ANOVA were used for overall comparisons using GraphPad Prism 6 (GraphPad Software, Inc., La Jolla, CA). Kruskal-Wallis test was used for nonparametric data. Corresponding post-*hoc* tests were used if there was an overall significance. *P* < 0.05 was considered to be significantly different.

### Data Availability Statement

The data generated during the current study are available from the corresponding author on reasonable request.

## Electronic supplementary material

Supplementary information
