# Supplementary material for: Effects of Intestinal Microbiota on Brain Development in Humanized Gnotobiotic Mice
Source: Sci Rep. 2018 Apr 3;8:5443. doi: 10.1038/s41598-018-23692-w (PMC5882882; doi:10.1038/s41598-018-23692-w)

Supplementary Information

Effects of Intestinal Microbiota on Brain Development in Humanized Gnotobiotic Mice

Jing Lu[1], Lei Lu[1], Yueyue Yu[1], Joanne Cluette-Brown[2], Camilia R. Martin[3], Erika C. Claud[1,*]

[1]The University of Chicago, Pritzker School of Medicine, Department of Pediatrics, Chicago, IL, 60637, USA
[2]Beth Israel Deaconess Medical Center, Division of Gastroenterology, Boston, MA, 02215, USA
[3]Beth Israel Deaconess Medical Center, Harvard Medical School, Department of Neonatology and Division of Translational Research, Boston, MA, 02215, USA
[*] eclaud@peds.bsd.uchicago.edu

Supplemental Figure S1

Blots with molecular markers illustrated in Figure 1.

Figure 1A

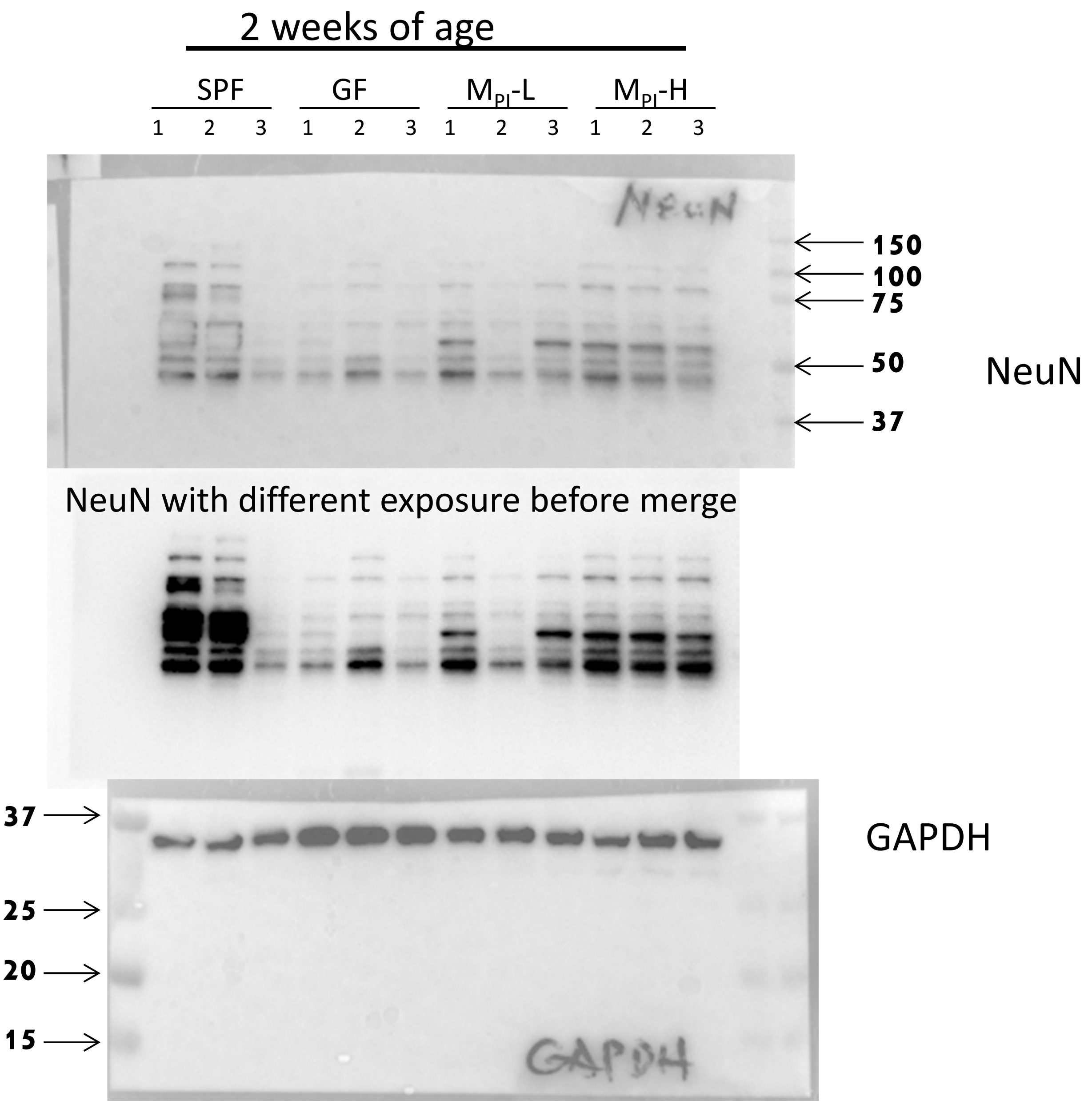

Supplemental Figure S1

Blots with molecular markers illustrated in Figure 1.

Figure 1B

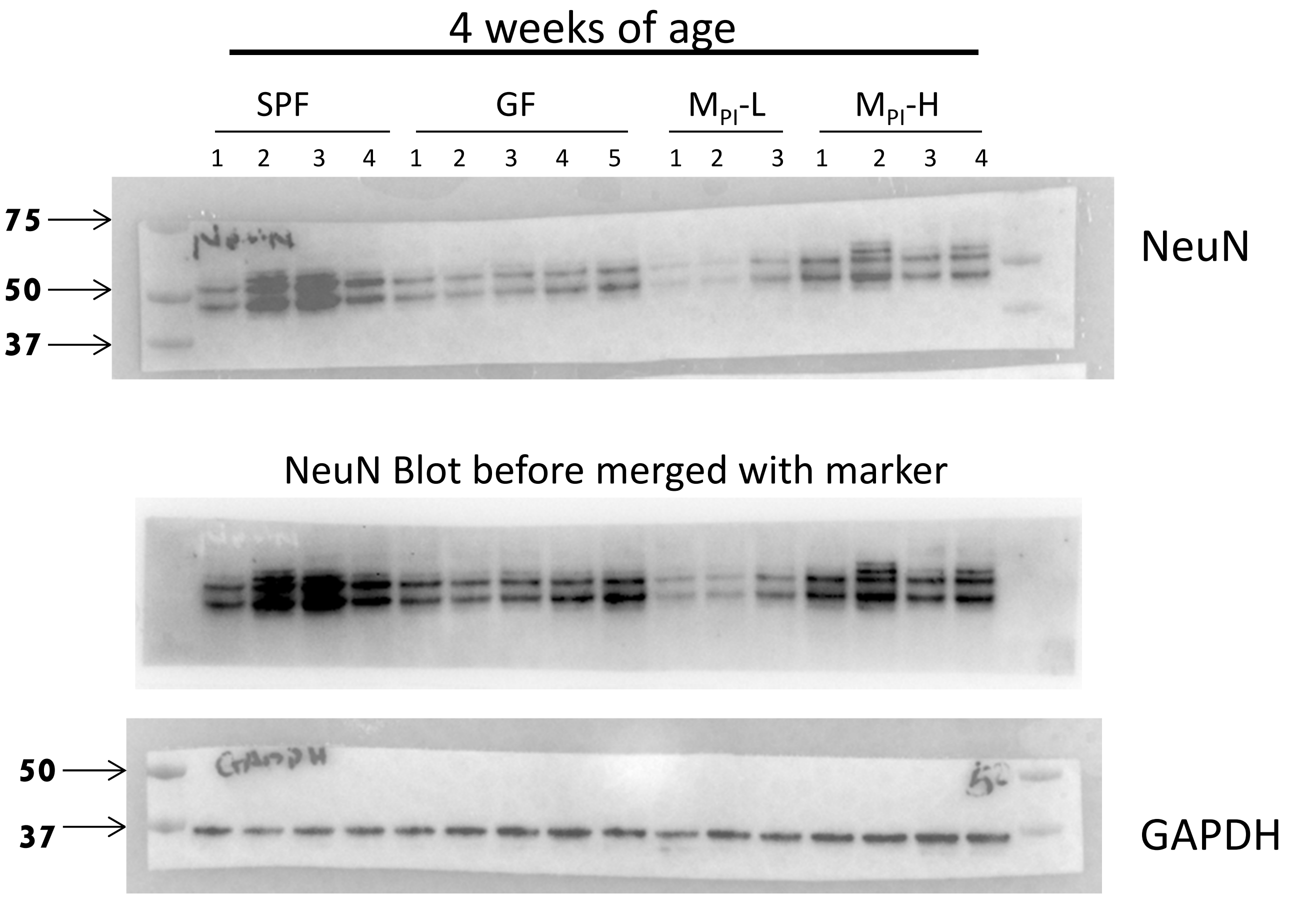

Supplemental Figure S1

Blots with molecular markers illustrated in Figure 1.

Figure 1E

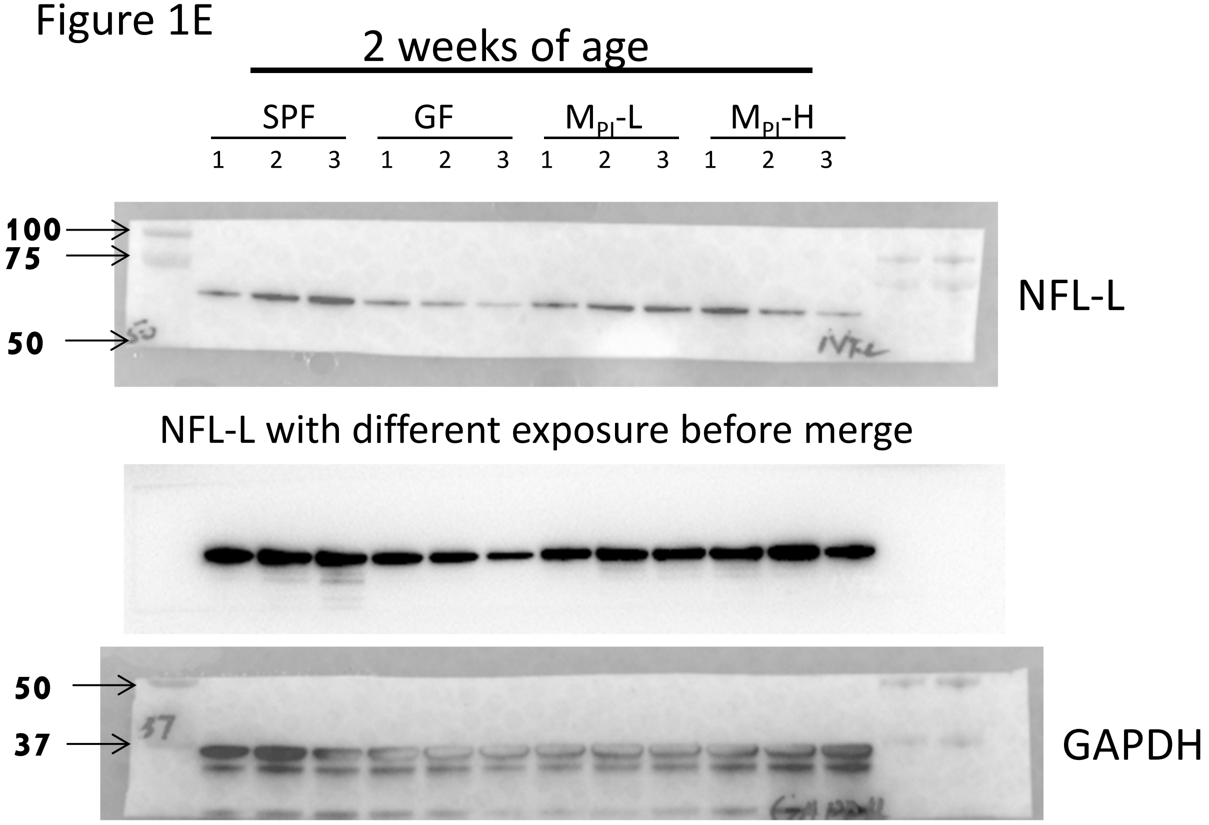

Supplemental Figure S1

Blots with molecular markers illustrated in Figure 1.

Figure 1F

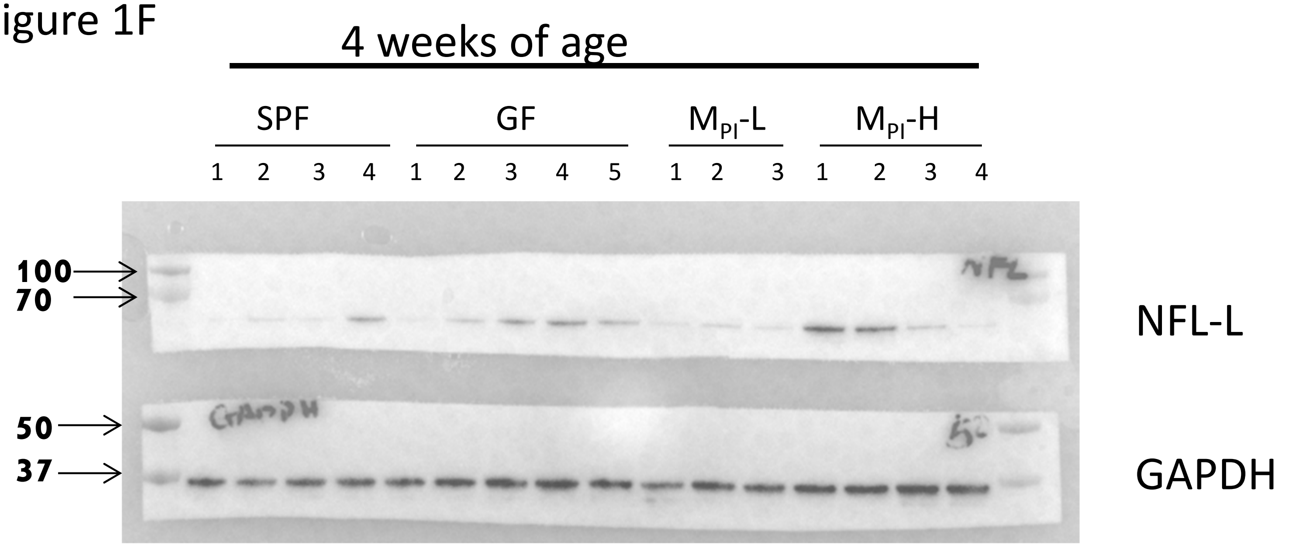

Blot with different exposure before merged with marker

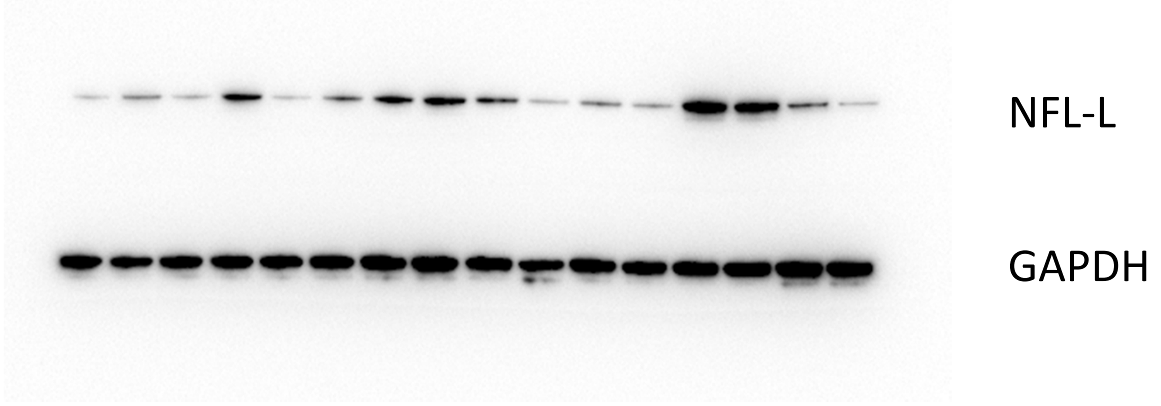

Supplemental Figure S2

Blots with molecular markers illustrated in Figure 2.

Figure 2A

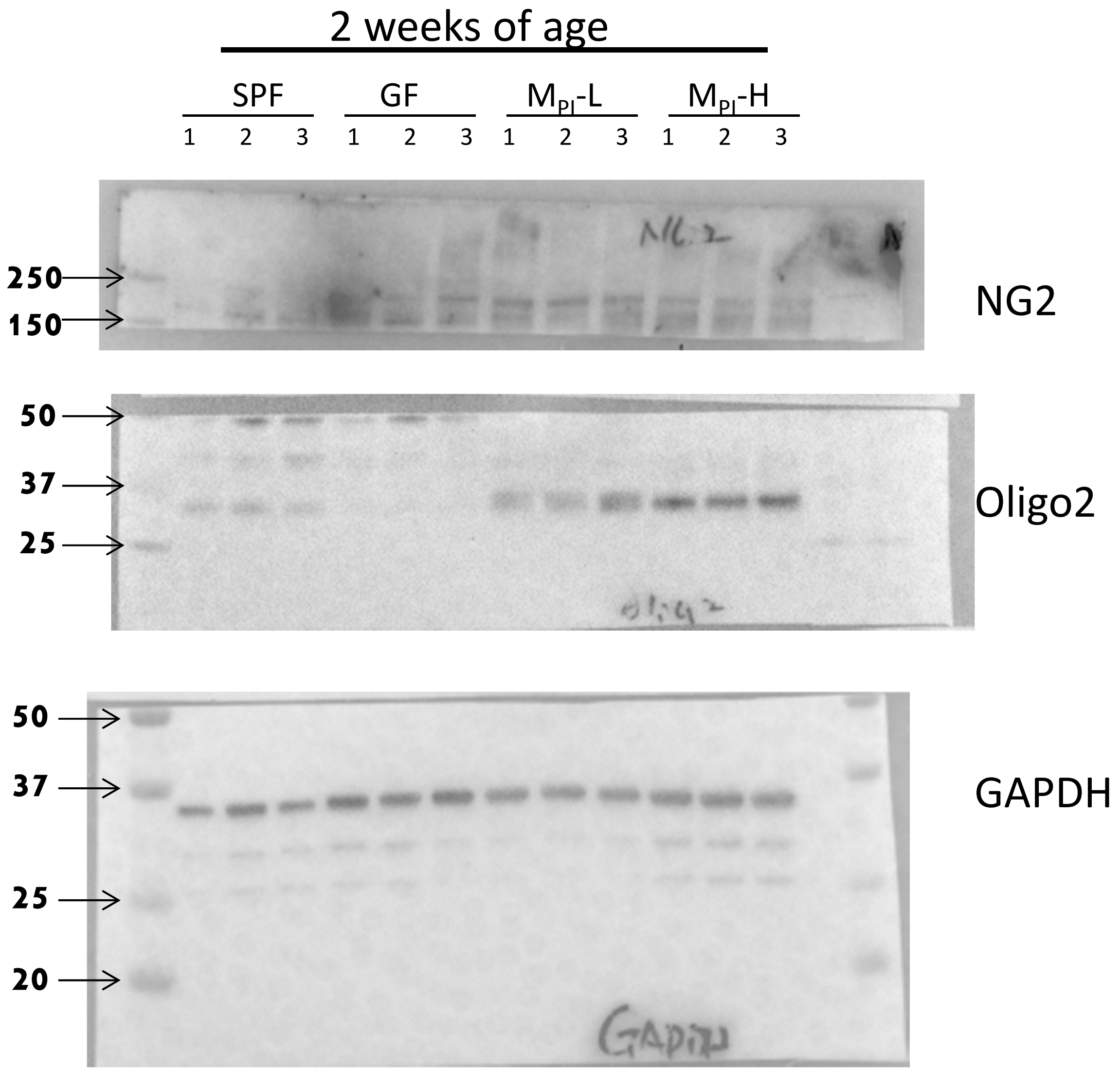

Supplemental Figure S2

Blots with molecular markers illustrated in Figure 2.

Figure 2B

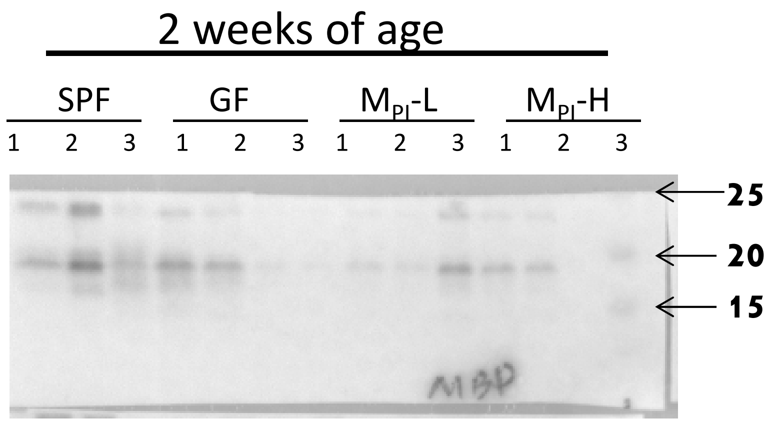

MBP

MBP Blot with different exposure before merged with marker

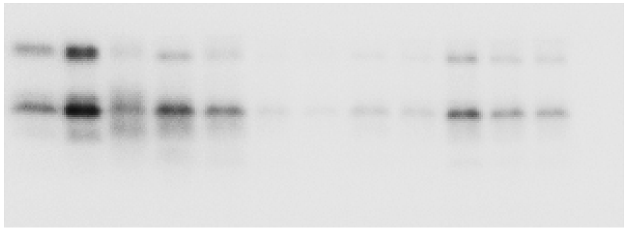

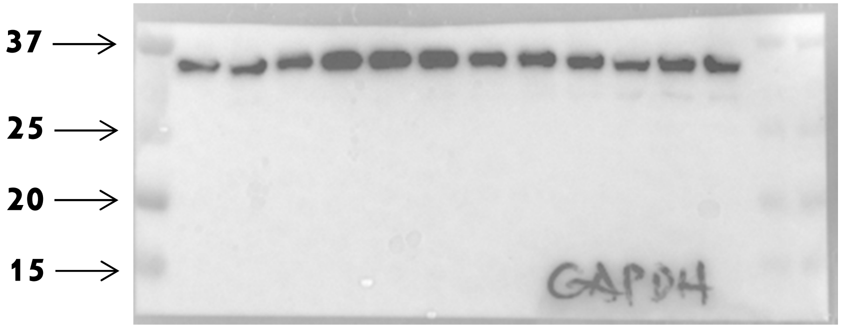

GAPDH

Supplemental Figure S2

Blots with molecular markers illustrated in Figure 2.

Figure 2C

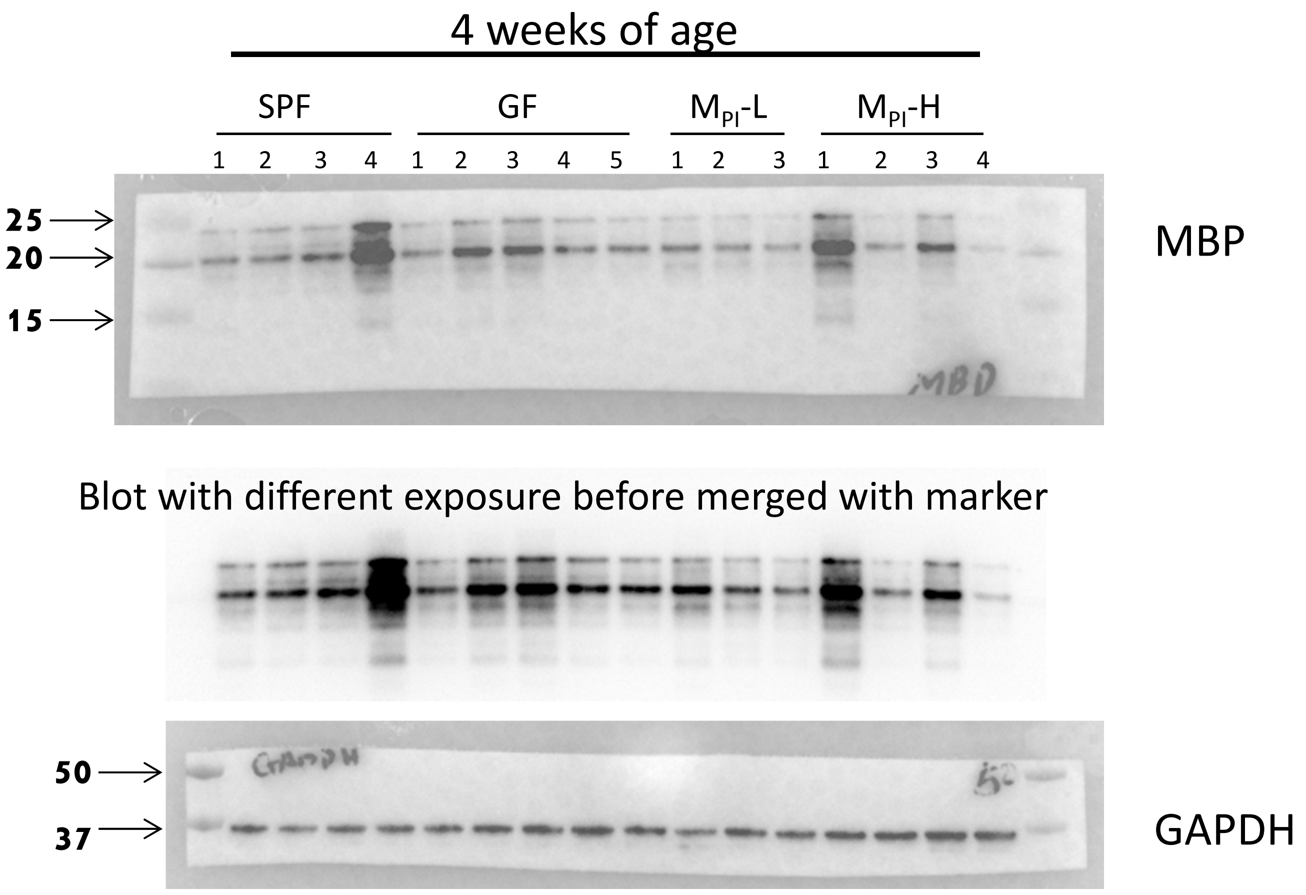

Supplement: Supplementary file 1 — Supplementary information [file 41598_2018_23692_MOESM1_ESM.pdf]
